# Parameter-Fitting-Free Continuum Modeling of Electric Double Layer in Aqueous Electrolyte

**DOI:** 10.1021/acs.jctc.4c00408

**Published:** 2024-07-05

**Authors:** Masao Suzuki Shibata, Yu Morimoto, Iryna V. Zenyuk, Adam Z. Weber

**Affiliations:** † Department of Chemical and Biomolecular Engineering and National Fuel Cell Research Center, University of California, Irvine, Irvine, California 92697, United States; ‡ Energy Conversion Group, Lawrence Berkeley National Laboratory, 1 Cyclotron Road, Berkeley, California 94720, United States

## Abstract

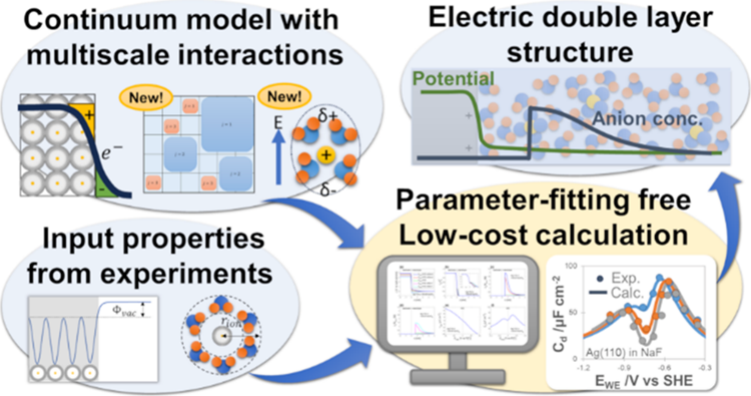

Electric double layers (EDLs) play fundamental roles in various electrochemical processes. Despite the extensive history of EDL modeling, there remain challenges in the accurate prediction of its structure without expensive computation. Herein, we propose a predictive multiscale continuum model of EDL that eliminates the need for parameter fitting. This model computes the distribution of the electrostatic potential, electron density, and species’ concentrations by taking the extremum of the total grand potential of the system. The grand potential includes the microscopic interactions that are newly introduced in this work: polarization of solvation shells, electrostatic interaction in parallel plane toward the electrode, and ion-size-dependent entropy. The parameters that identify the electrode and electrolyte materials are obtained from independent experiments in the literature. The model reproduces the trends in the experimental differential capacitance with multiple electrode and nonadsorbing electrolyte materials (Ag(110) in NaF, Ag(110) in NaClO_4_, and Hg in NaF), which verifies the accuracy and predictiveness of the model and rationalizes the observed values to be due to changes in electron stability. However, our calculation on Pt(111) in KClO_4_ suggests the need for the incorporation of electrode/ion-specific interactions. Sensitivity analyses confirmed that effective ion radius, ion valence, the electrode’s Wigner–Seitz radius, and the bulk modulus of the electrode are significant material properties that control the EDL structure. Overall, the model framework and findings provide insights into EDL structures and predictive capability at low computational cost.

## 1 Introduction

The electric double layer (EDL) plays a fundamental role in various electrochemical processes, such as colloidal dispersions, surface charging, and charge-transfer reactions. It appears at every electrode/electrolyte interface because of the work function difference between the two materials. The work function difference induces a strong electric field at the interface. The electric field redistributes the solute and solvent molecules in the electrolyte and forms a surface charge and dipole by either attracting or repulsing the molecules to or from the electrode surface. At the same time, electrons in the electrode also redistribute to form a surface charge and dipole by spilling into the electrolyte.^1^ The surface charge due to the electron spillover is the same value in the opposite sign as that due to the redistribution of the molecules in the electrolyte, which ensures overall electroneutrality. The layered structure formed by the redistribution of electrons in the metal and molecules in the electrolyte is called the EDL.

The increasing focus of EDL studies lies in its impact on reaction kinetics. The electric field strength and electrostatic potential near the interface are determined by the EDL structure, which can impact the concentration and stability of the reactants. Ringe et al.^2^ and Shin et al.^26^ suggested that the electric field strength at the interface can impact the stability of CO_2_ adsorbate and thus the reaction kinetics of CO_2_ reduction. Similar effects of EDLs on the reaction kinetics can appear in any catalytic reactions in electrochemical systems. A deeper understanding of these effects will provide guidance for tailoring the electrode/electrolyte interface with improved reaction kinetics.

Differential capacitance (*C*_d_) has been analyzed as a fundamental property of EDLs.^3−5^*C_d_* is defined as *C*_d_ ≔ d*Q*/d*V*, where *Q* is the surface charge density and *V* is the electrode potential. This property represents the potential dependence of the ion distribution in the electrolyte. *C*_d_ can be experimentally obtained by cyclic voltammetry (CV) or electrochemical-impedance spectroscopy (EIS). In CV measurements, however, separating EDL capacitance from total capacitance can be challenging when a Faradaic reaction occurs in the potential range of interest.^6^ EIS measurements with appropriate analysis can separate those two currents based on the difference in their time scales.^4^

As a continuum modeling approach, Gouy–Chapman–Stern (GCS) theory has been widely used as a standard model for analyzing EDL structures.^1,5,7−10^ This model envisions the EDL as two separate layers: Stern layer and diffuse layer. The Stern layer is a layer between the electrode surface and the closest approach of hydrated ions. In this layer, hydrated ions cannot exist because of the size exclusion. Without specific ion adsorption, the Stern layer does not contain charged species. The dielectric constant in the Stern layer can be much smaller than that of the bulk solvent (∼an order of magnitude) due to impacts of water structure^11−15^ and field effects^16−18^ near the interface. Unlike in the Stern layer, ions can redistribute, depending on the electrostatic potential in the diffuse layer. The distribution is obtained from the Poisson equation and the conditions for thermodynamic equilibrium. The analytical solution for the ion distribution is called Gouy–Chapman distribution when the chemical potential of ions (μ*_i_*) is described in the form of
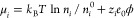
1where *k*_B_ is the Boltzmann constant, *T* is the temperature, *n_i_* is the number density of solute *i*, *n*_i_^0^ is the number density of *i* in the bulk, *z*_*i*_ is the ionic valency, *e*_0_ is the elementary charge, and ϕ is the electrostatic potential. Bikerman^19^ improved the accuracy of the diffuse-layer model by modifying the entropy term (first term on the right side) to account for the finite size of the ions. He obtained an analytical expression for the entropy term by assuming that all of the ions in the electrolyte have the same molar volume.^20^ The continuum model based on GCS theory with Bikerman modification is widely used as a primitive EDL model because of its computational efficiency.

Conventional continuum models, however, are not capable of “predicting” the EDL structure by themselves. They require parameter fitting, especially for the parameters in the Stern layer. The shortcomings of the conventional continuum models stem from the fact that they do not explicitly account for several fundamental microscopic interactions near the interface^21^ such as electron spillover and ion-specific adsorption.

Quantum-mechanical calculations like density functional theory (DFT)^22−24^ provide the means for predictive analyses of the interface. DFT calculations capture the effect of electron spillover, as well as the specific interactions between the electrode/solute, electrode/solvent, and solute/solvent. The analysis can be further improved by using *ab initio* molecular-dynamics simulations (AIMDs).^25,26^ AIMDs can include quantum-mechanical interactions, as well as steric restrictions and entropic contributions. However, because of their large computational cost to evaluate the average structure of the statistical equilibrium, these calculations are limited to a small volume that is typically not sufficient to capture the whole picture of the EDL structure accurately.

The advantages of continuum models and quantum-mechanical calculations can be achieved by coupling these two models. Joint density functional theory (joint DFT) was proposed to combine the quantum DFTs and classical continuum model.^27,28^ Huang proposed a simple and effective model named density potential functional theory (DPFT).^20^ This model combines a classical continuum model based on GCS theory with a Bikerman modification and a simplified quantum-mechanical calculation. For the quantum-mechanical model, he employed a jellium model with uniform electron gas approximation.^22^ A jellium model, which describes the positive charge distribution in the electrode as a uniform background charge, is one of the simplest models that accounts for the spillover of electrons into the electrolyte. Although the jellium model does not provide results with highest accuracy, it has been used for work function analyses^29−33^ and EDL modeling^34−36^ due to its computational efficiency. The accuracy of a jellium model can be improved by using pseudopotentials.^30−33^ Even with Huang’s DPFT model,^20^ however, parameter fitting is needed to reproduce the experimental differential capacitance measurements.

This study proposes a new modeling framework that gains the advantages of both continuum models and molecular-dynamics simulations, *i*.*e*., accurate and predictive analyses with low computational cost. This model computes the distribution of the electrostatic potential, electron density, and species’ concentrations by taking the extremum of the total grand potential of the system. The grand potential is calculated from the entropic energy, electrostatic energy, electron energy, solute–solute interactions, and electrode/solute interactions. These energy expressions include not only the terms from previous papers^20^ but also the microscopic interactions that are newly introduced in this work: polarization of solvation shells, electrostatic interaction in parallel plane toward the electrode, and ion-size-dependent entropy. All parameters that identify the electrode and electrolyte materials are obtained from independent experiments; in this way, the model is predictive and does not contain additional fitting parameters.

## 2 Theory

### 2.1 General Approach

The EDL is often assumed to be in a quasi-equilibrium^37^ because of its rapid formation. The formation time scale can be roughly expressed as 10 *l*_D_^2^/*D*, where *l*_D_ is the Debye length and *D* is the diffusion coefficient,^38^ and can scale from 10^–9^ to 10^–6^ s for 1 M to 1 mM of 1:1 electrolytes with a diffusion coefficient of 10^–9^ m^2^/s, which is much shorter than time scales of typical electrochemical measurements (*e*.*g*., EIS measurement with 100 kHz corresponds to the time scale of 10^–5^ s).^1,7^ Based on the time scale difference, it is reasonable to assume that EDL is in quasi-equilibrium. Also, we focus on potential windows in which the Faradaic reaction rate is small so that the chemical potential gradient of solutes in EDL is negligible. Based on these assumptions, the distribution of electrostatic potential (ϕ), electron density (*n*_e_), and species’ number density (*n*_*i*_) are obtained by taking the extremum of the total grand potential (Ω_tot_) of the system.^20,37^ More specifically, *n*_e_ and *n*_*i*_ are obtained by minimizing Ω_tot_, while ϕ is obtained by maximizing it.^39^ Ω_tot_ can be expressed as a functional of these unknown variables (ϕ, *n*_e_, and *n*_*i*_) as
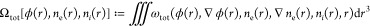
2where ω_tot_ is the local grand potential. Here, we assume the electrode is flat, and ϕ, *n*_e_, and *n*_*i*_ distribute uniformly in the plane parallel to the electrode. With this assumption, the grand potential can be simplified in one-dimension (1D) to
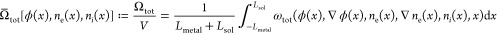
3where *x* describes the position in the direction perpendicular to the electrode. *x* < 0, *x* = 0, and *x* > 0 represent the electrode, electrode/electrolyte interface, and electrolyte, respectively. *V* is the volume of the system. *x* = −*L*_metal_ is the left end of the calculation domain that represents the bulk metal, and *x* = *L*_sol_ is the right end of the domain that represents the bulk solution. The local grand potential can be decomposed into 5 factors.
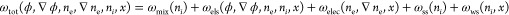
4where ω_mix_ is the mixing entropy, ω_els_ is the electrostatic energy, ω_elec_ is the electron energy, ω_ss_ is the solute–solute interaction, and ω_ws_ is the wall (electrode)-solute nonelectrostatic interaction. The reference potentials of all the grand potential components are based on the electrochemical potential in the bulk electrolyte (denoted by the superscript 0) so that the conditions to take the extremum of Ω̅_tot_, which is described in the next section, are satisfied in the bulk. The expressions for ω_tot_ are shown in Section 2.3 and listed in Tables S1–S3 in the Supporting Information (SI).

### 2.2 Variational Analysis

ϕ(*x*), *n*_e_(*x*), and *n*_*i*_(*x*) are obtained from the condition to let Ω̅_tot_[ϕ(*x*), *n*_e_(*x*),*n*_*i*_(*x*)] be the extremum. The condition can be written as δΩ̅_tot_/δϕ(*x*) = δΩ̅_tot_/δ*n*_e_(*x*) = δΩ̅_tot_/δ*n*_*i*_(*x*) = 0 for any *x*. By considering that the local grand potential ω_tot_ depends not only on ϕ, *n*_e_, and *n*_*i*_, but also on the first-order gradient of ϕ and *n*_e_ (∇ϕ and ∇*n*_e_), the conditions are expressed as
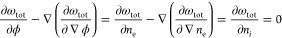
5where ϕ, *n*_e_, and *n*_*i*_ need to satisfy these equations at any position of *x*. Equation 5 provides the same number of equations as the number of unknown variables (ϕ(*x*), *n*_e_(*x*), and *n*_*i*_(*x*)). Hence, the distributions of the variables are determined by numerically solving these equations. For the numerical calculations, we used COMSOL Multiphysics version 6.1. One should note that the condition for ϕ corresponds to the Poisson equation, the condition for *n*_e_ corresponds to the electron density model in jellium model, and the condition for *n*_*i*_ corresponds to the thermodynamic equilibrium equation. The formulas for the partial differential equations (PDEs) derived from eq 5 are shown in Section S3 in the SI.

### 2.3 Local Grand Potential Expressions

#### 2.3.1 Mixing Entropy, ω_mix_

Bikerman^19^ proposed a model for the mixing entropy that accounts for the finite size of the species. This expression is frequently employed in continuum models for EDLs because of the availability of analytical solutions. However, the model is associated with the total free energy by assuming that all the species in the solution have the same molar volume,^40−42^ which is not always applicable to ions with different molecular size or hydration numbers.

There are several existing models that deal with the asymmetric size of the solute molecules. For example, Gongadze and Iglic^43^ proposed a model by modifying the Bikerman model, providing an analytical expression for solute concentrations with molecules' size variation. Another model proposed by Maggs and Podgornik^44^ accounted for the entropy due to the solvent. However, a comprehensive formulation of mixing entropy with an asymmetric size effect has yet to be established. The Gongadze’s model involves an unphysical segmentation of the solutes to achieve uniform size, while the Maggs’ model solely accounts for solvent entropy, neglecting entropy loss of solutes due to the existence of other solutes.

Hence, we derived the mixing entropy (ω_mix_) based on the partition function with a lattice model,
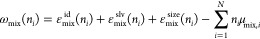
6where ε_mix_^id^ is the entropy from ideal mixing, ε_mix_^slv^ is the solvent’s entropy considering the finite size of the solute molecules, and ε_mix_^size^ is the entropy due to the difference in the species’ molar volume. μ_mix,*i*_ is the chemical potential due to the mixing in the bulk solution that is defined to make sure ∂ω_mix_/∂*n*_*i*_ = 0 in the bulk. This mixing entropy is based on the partition function using a lattice model as
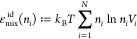
7
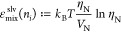
8
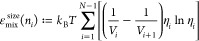
9
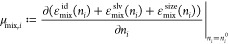
10where *N* is the number of the solute type, *V*_*i*_ is the volume of one molecule of species *i*, and . Smaller *i* represents larger solute molecule so that *V*_1_ ≥ *V*_2_ ≥ ··· ≥ *V*_*N*_. The derivation of these equations is shown in Section S2.1 in the SI. When all of the solute molecules have the same molar volume, this expression reduces to the conventional Bikerman model.^19^

#### 2.3.2 Electrostatic Interactions, ω_els_

The grand potential due to electrostatic interactions can be expressed by
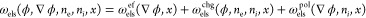
11where ω_els_^ef^ is the electric field energy, ω_els_^chg^ is the Coulombic energy of charged species, and ω_els_^pol^ is the polarization energy of solvent and solute molecules. In this study, ω_els_^ef^ and ω_els_^chg^ use conventional expressions based on the Coulombic interactions.^37^
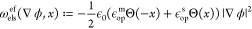
12
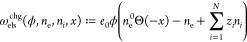
13where ϵ_0_ is the vacuum permittivity, *e*_0_ is the elementary charge, and *n*_e_^0^ is the valent electron density in the bulk metal. Θ(*x*) is the Heaviside step function which takes a value of 1 when *x* > 0 and 0 when *x* < 0. Θ(*x*) is used to express the metal properties in *x* < 0 and solution properties in *x* > 0. ϵ_op_^m^ and ϵ_op_^s^ are the optical dielectric constant of the metal and solvent, respectively. ω_els_^pol^ accounts for the dielectric saturation^16,17^ and the polarizability of hydrated ions.^45,46^ In the calculation, we assume that dielectric saturation of ion solvation shell takes place in a similar way to water molecules^17,46^
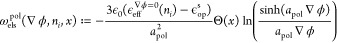
14where *a*_pol_ is a constant that controls the significance of dielectric saturation: large *a*_pol_ increase the effect of electric field on the effective dielectric constant. *a*_pol_ is obtained by fitting to an *ab initio* molecular dynamic analysis.^17^ ϵ_eff_^∇ϕ = 0^ is the dielectric constant without electric field and is defined as , where β_*i*_ is the polarizability of solute *i*, ϵ_s_^0^ is the solvent’s dielectric constant, and *N*_avo_ is the Avogadro’s constant. The electrostatic potential in the bulk solution is set to zero. With these expressions of ω_els_, δΩ̅_tot_/δϕ(*x*) = 0 gives the Poisson equation with the effective dielectric constant ϵ_eff_ as described in eqs S31 and S32 in the SI. In the bulk solution (|∇ϕ| → 0, *x* > 0, *n*_*i*_ = *n*_*i*_^0^), the effective dielectric constant becomes *ϵ*_eff_ = *ϵ*_eff_^∇ϕ=0^(*n*_*i*_^0^) = . Hence, β_*i*_ can be obtained by measuring concentration-dependent dielectric constant as β_*i*_ = *–N*_avo_dϵ_eff_/d*n*_*i*_^0^^45^ for the bulk solution.

#### 2.3.3 Electron Energy, ω_elec_

This study employs electron energy to account for the effect of a potential-dependent electron spillover. We employ a jellium model with uniform electron gas approximation^22^ for its simple and low-cost computation. In the jellium model, positive charges in core atoms of the metal (*n*_m_) are expressed as the uniform background charge, and the density distribution of the valence electrons (*n*_e_(*x*)) is evaluated based on the kinetic and exchange-correlation energy of electrons with the mean-field approximation. Based on Smith’s expression,^29^ our model evaluates the kinetic energy with the first-order density-gradient expansion, while it uses the local-density approximation for exchange-correlation energy. Because of the simplified interaction between the core electrons and the valence electrons, jellium models need modifications to improve their accuracy. Perdew et al.^32^ introduced a structureless pseudopotential that represents the Madelung energy and the repulsive interaction from the core electrons. The accuracy of Perdew’s model in predicting the work function and the bulk modulus of metals is, however, limited mainly because of the assumption of uniform background positive charge in the jellium model. The discrepancy becomes larger when the model is applied to transition and noble metals. Russier and Badiali attributed the discrepancy on transition and noble metals to the contribution of d-electron.^47^ To correct the errors, we employ the structureless pseudopotential in a semiempirical manner. We employ two parameters in the model that are determined based on basic metal properties. One parameter, structureless pseudopotential^30,32^ (μ_ps_^m^), was determined from the bulk modulus of the metal (see eq S29 in the SI). The other parameter, Δϕ_WF_, was determined from the work function in vacuum and assumed to be independent of the electrode potential (see Section 2.5.1). We also assumed that the electrons that spill into the electrolyte phase feel a non-negligible potential due to the interaction with solvent molecules. This potential was expressed as the pseudopotential in the electrolyte phase. It was determined from the potential of zero charge of Ag(110)^48^ and used as a constant value for other metals.

The grand potential for the electron energy is expressed as
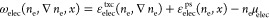
15where ε_elec_^txc^ is the summation of kinetic, exchange, and correlation energy of the electron based on Smith’s expression^29^
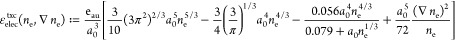
16where *e*_au_ is the Hartree energy and *a*_0_ is the Bohr radius. ε_elec_^ps^ is the structureless pseudopotential that takes different values in the metal (*x* < 0) and in the solution (*x* > 0),
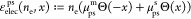
17where μ_ps_^m^ and μ_ps_^s^ are the pseudopotentials in the metal and solution, respectively, and μ_elec_ is the chemical potential of the electron and is defined as
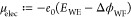
18where *E*_WE_ is the absolute potential of the working electrode and Δϕ_WF_ is a constant used to correct the potential to match the vacuum work function. *E*_WE_ is the control parameter in the calculation.

#### 2.3.4 Solute–Solute Interaction, ω_ss_

We account for the electrostatic solute–solute interactions by assuming that the solvation shells prevent the solutes from getting close to other solute molecules to feel chemical interactions. Although the electrostatic interaction (ω_els_) accounts for the electrostatic interaction along the *x* direction; it does not include the interaction in the *y*–*z* plane because it is a one-dimensional model. Ions in the solution interact with each other and redistribute due to electrostatic interaction, which affects the total energy. This *y*–*z* plane electrostatic interaction is accounted for as solute–solute interaction. The solute–solute interaction is obtained by analytically solving the Poisson–Boltzmann equation. The governing equations are the same as that of Debye–Hückel theory; however, we used a cylindrical coordinate in the *y*–*z* plane, instead of spherical coordinates, to avoid double counting electrostatic interactions in the *x* direction. The interaction is thus expressed in the form of
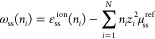
19where ε_ss_^ion^ is the ion–ion interaction in the *y*–*z* plane and μ_ss_^ref^ is the reference chemical potential expressed as
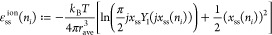
20
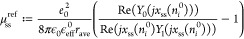
21where *r*_ave_ is the average radius of the solute molecules, *j* is the imaginary unit, *Y*_0_ and *Y*_1_ are the Bessel functions of the second kind of order 0 and order 1, respectively, and *x*_ss_ is an intermediate variable described by *x*_*ss*_(*n*_*i*_) ≔ . In the derivation of this expression, we employed the first-order approximation |*z*_*i*_*e*_0_ϕ/*k*_B_*T*| ≪ 1. Although this approximation becomes less accurate when the ion effective radius is small, it enables analytic expression, which is needed to include the effect of *y*-*z* direction distribution in the one-dimensional grand potential, ω_tot_. This interaction is newly introduced in this model, whose derivation is shown in Section S2.2 in the SI.

#### 2.3.5 Wall-Solute/Solvent Interaction, ω_ws_

To simplify the analysis, this work focuses on a system without a significant specific interaction between the electrode and solute or solvent. Hence, in this study, ω_ws_ only accounts for solute ions’ steric restriction for closest approach due to their finite size
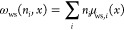
22

where μ_ws,*i*_ is the chemical potential for wall-solute/solvent interactions and is defined as μ_ws,*i*_:= μ_cut_Θ(*r*_*i*_ – *x*), μ_cut_ is the cutoff chemical potential (set to 1000 eV), and *r*_*i*_ is the species’ effective radius.

### 2.4 Boundary Conditions

Since the problem to be solved is one-dimensional with physics described by second-order differential equations, we need two boundary conditions for each variable. For electrostatic potential, the boundary conditions are set as ϕ(*L*_sol_) = 0 and ∇ϕ(*−L*_metal_) = 0. For electron density, *n*_e_(*L*_sol_) = 0 and *n*_e_(*−L*_metal_) = *n*_e_^0^. From the definition of the reference energy, ∇*n*_e_(*L*_sol_) = ∇*n*_e_(*−L*_metal_) = 0 is satisfied when *L*_slv_ and *L*_metal_ are large enough. For species’ density *n*_*i*_, the boundary conditions are ∇*n*_*i*_(*L*_sol_) = ∇*n*_*i*_(0) = 0, which automatically satisfies *n*_*i*_(*L_sol_*) = *n*_*i*_^0^ because of the definition of the reference energy. With these boundary conditions, the working electrode (*E*_WE_) was controlled to estimate how the system reacts with the electrode potential change.

### 2.5 Parameter Setting

Here we describe the parameter setting protocols for electrode- or electrolyte-specific parameters. The values of the parameters for the electrode and electrolyte are listed in Tables 1 and 2, respectively.

**Table 1:** Input Properties of the Electrodes^a^

	*r*_ws_/a.u.	*B*/Mbar	Φ_vac_^exp^/eV
Ag(110)	3.01 (ref (49))	1.01 (ref (33))	4.14 (ref (51))
Pt(111)	2.90 (ref (49))	2.78 (ref (33))	5.93 (ref (52))
Hg	3.10 (ref (49))	0.267 (ref (50))	4.48 (ref (53))

a The calculated value for Δϕ_WF_ to satisfy the vacuum work function (Φ_vac_^exp^) are 1.40, 3.01, and 1.32 V for Ag(110), Pt(111), and Hg, respectively.

**Table 2:** Input Properties of the Electrolyte

	*r*_*i*_/nm (ref (54))^a^	β_*i*_/M^–1^ (ref (45))	*z*_*i*_
Na^+^	0.65	7	+1
K^+^	0.69	10	+1
F^–^	0.40	3	–1
ClO_4_^–^	0.51	1	–1

a For anions, only the first hydration shell was included, while second hydration shell was included for cations assuming the stronger hydration affinity of cations.^55^ For water radius, 0.138 nm was used.^54^

#### 2.5.1 Electrode-Specific Parameters

The model uses three electrode-specific experimental properties: the Wigner–Seitz radius (*r*_ws_),^49^ the bulk modulus (*B*),^33,50^ and work function in the vacuum (Φ_vac_^exp^).^51−53^ The Wigner–Seitz radius is employed to evaluate the electron density in the bulk metal, *n*_e_^0^:= 3/(4π*r*_ws_^3^). The bulk modulus is used to estimate the pseudopotential in the metal (μ_ps_^m^) using the relation *B* = *V*(∂^2^*E*)/(∂*V*^2^)*_N_*, where *V*, *E*, and *N* are the volume, energy, and number of electrons in the metal, respectively (see Section S2.3 in the SI). Δϕ_WF_ was assumed to be a constant and was determined from the work function in vacuum as Δϕ_WF_ = Φ_vac_^exp^ – Φ_vac_^calc^, where Φ_vac_^exp^ is the experimental work function and Φ_vac_^calc^ is the calculated work function when Δϕ_WF_ = 0. In the calculation of work function in vacuum, we set the effective dielectric constant to be 1, species’ density *n*_*i*_ to be 0, and pseudopotential outside of the metal to be 0.

#### 2.5.2 Electrolyte-Specific Parameters

The model employs six electrolyte-specific properties: the ionic valence of the anion and cation (*z*_a_ and *z*_c_), effective hydrated radius of anion and cation (*r*_a_ and *r*_c_), and the polarizability of the hydrated shell of anion and cation (β_a_ and β_c_). *z*_*i*_ is obtained from the ionic formula. β_*i*_(:= d*ϵ*_eff_/d*c*_*i*_) is from the experimental results for concentration-dependent dielectric potential.^45^*r*_*i*_ is calculated from the distance between the ion’s center and center of the nearest water molecule,^54^ including up to the first water layer for anions and second water layer for cations.^55^ The electrons’ pseudochemical potential in the solvent (μ_ps_^s^) also needs to be determined using an experimental result. We employed the potential of zero charge (PZC) on Ag(110) in 5 mM NaF (−0.731 V vs SHE^48^) to get μ_ps_^*i*^ = −0.043 eV. We fixed this value for all of the calculations using aqueous electrolyte because we assume that it stems from the interaction between the electron from metal and the water molecules.^36^ In the calculation, the absolute potential for standard hydrogen electrode (SHE) is set as 4.44 V.^56^

## 3 Results and Discussion

### 3.1 Evaluation of the Calculated Results

**Figure 1:**
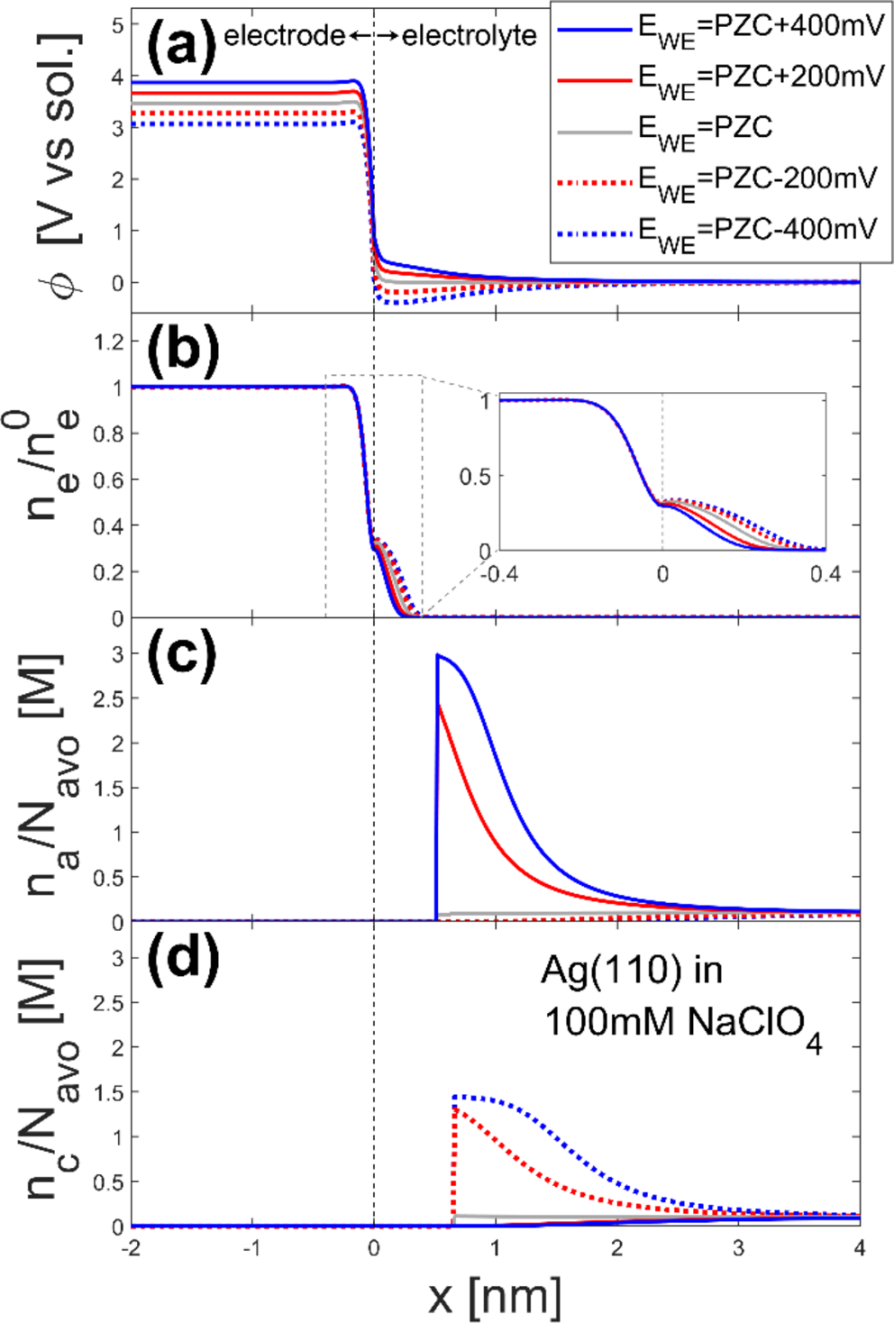
Calculated distributions of (a) electrostatic potential (ϕ), (b) electron density (*n*_e_), (c) anion density (*n*_a_), and (d) cation density (*n*_c_) for Ag(110) in 100 mM NaClO_4_. The horizontal axis shows the position in the *x* direction (*x* < 0 is in the electrode phase and *x* > 0 is in the electrolyte phase), and the different line styles represent different electrode potentials as shown in the legend in (a). The inset in (b) is the enlarged view of the domain inside the gray dashed box. *N*_avo_ is the Avogadro number. The black dashed line indicates the interface between the electrode (*x* < 0) and the electrolyte (*x* > 0).

**Figure 2:**
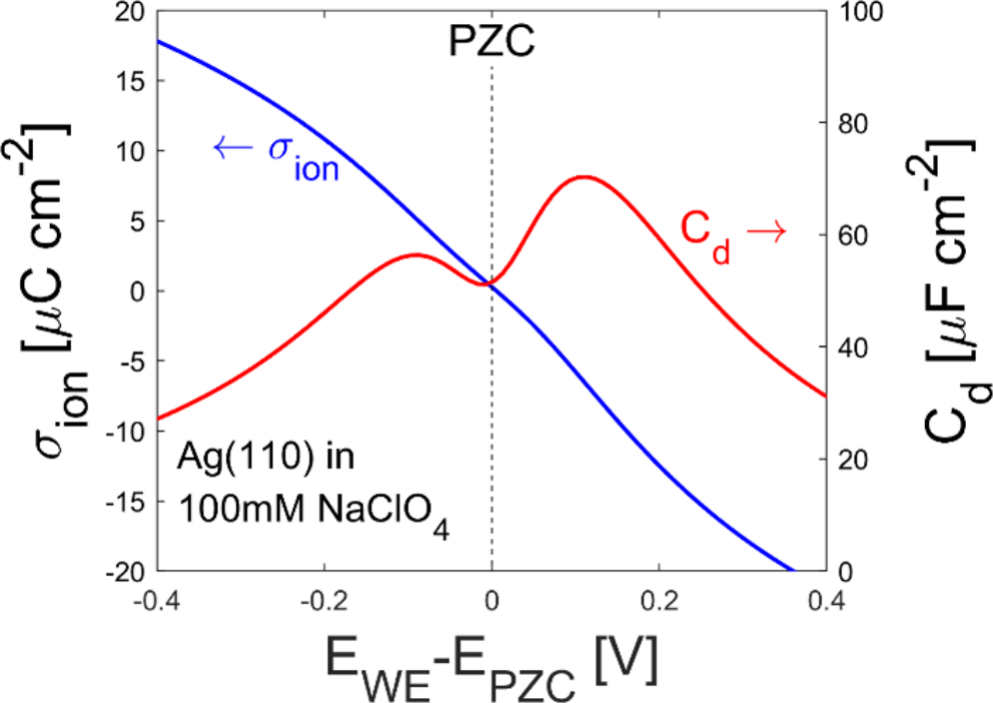
Calculated surface charge density of ions (σ_ion_, blue line, left axis) and differential capacitance, (*C*_d_, red line, right axis) with respect to the electrode potential (*E*_WE_). The parameter set for Ag(110) in 100 mM NaClO_4_ is used. The black dashed line corresponds to the potential of zero charge (PZC). σ_ion_, *E*_WE_, and *E*_PZC_ represent the surface charge in the electrolyte (eq 23), the working electrode potential, and the PZC, respectively.

The model calculates the distribution of ϕ, *n*_e_, and *n*_*i*_ (cations: *i =* c, anions *i =* a) at the given electrode potential (*E*_WE_) as shown in Figure 1. The material-specific parameters are listed in Tables 1 and 2. The electrostatic potential (ϕ) increases under a higher electrode potential, while the electron density (*n*_e_) in the electrolyte phase decreases for higher *E*_WE_. The anion density (*n*_a_) is higher when a higher potential is applied, while the opposite trend is observed for cations (*n*_c_). The anion and cation density decrease to almost zero, respectively, at *x* ≤ 0.51 nm and *x* ≤ 0.65 nm because of the steric wall-solute interaction (ω_ws_) defined in eq 22, corresponding to the Stern layer. These trends are physically representative and reasonably consistent with the previous analyses for the EDLs.^8,9,20^ By integrating the charge density of ions in the electrolyte phase, one can obtain the surface charge density σ_ion_
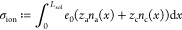
23when *L*_sol_ and *L*_metal_ are large, the total charge density σ_tot_:= σ_ion_ + σ_metal_ becomes zero, where
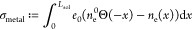
24The first term in the parentheses represents the positive background charge in the metal. At a potential of zero charge (PZC), σ_ion_ = σ_metal_ = 0. For comparison with the experiment, the differential capacitance, *C*_d_, was calculated by taking the derivative of σ_ion_ with respect to the electrode potential
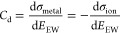
25The differential capacitance is experimentally measurable with EIS or CV by using the relation of dσ_ion_/d*t* = −*I*, where *I* is the current (positive current means an oxidative current). The surface charge density and differential capacitance are shown in Figure 2. The surface charge density is positive below the PZC and negative above the PZC. In the varied potential range, the surface charge density is monotonic. The double-layer capacitance exhibits a local minimum at the PZC because this is where the interface is least charged, and then there are two humps below and above PZC that are not symmetric. This asymmetry arises from anions being smaller in effective hydrated radius compared to the cations, which is discussed in Section 3.3.

### 3.2 Comparison with Experimental Results

**Figure 3:**
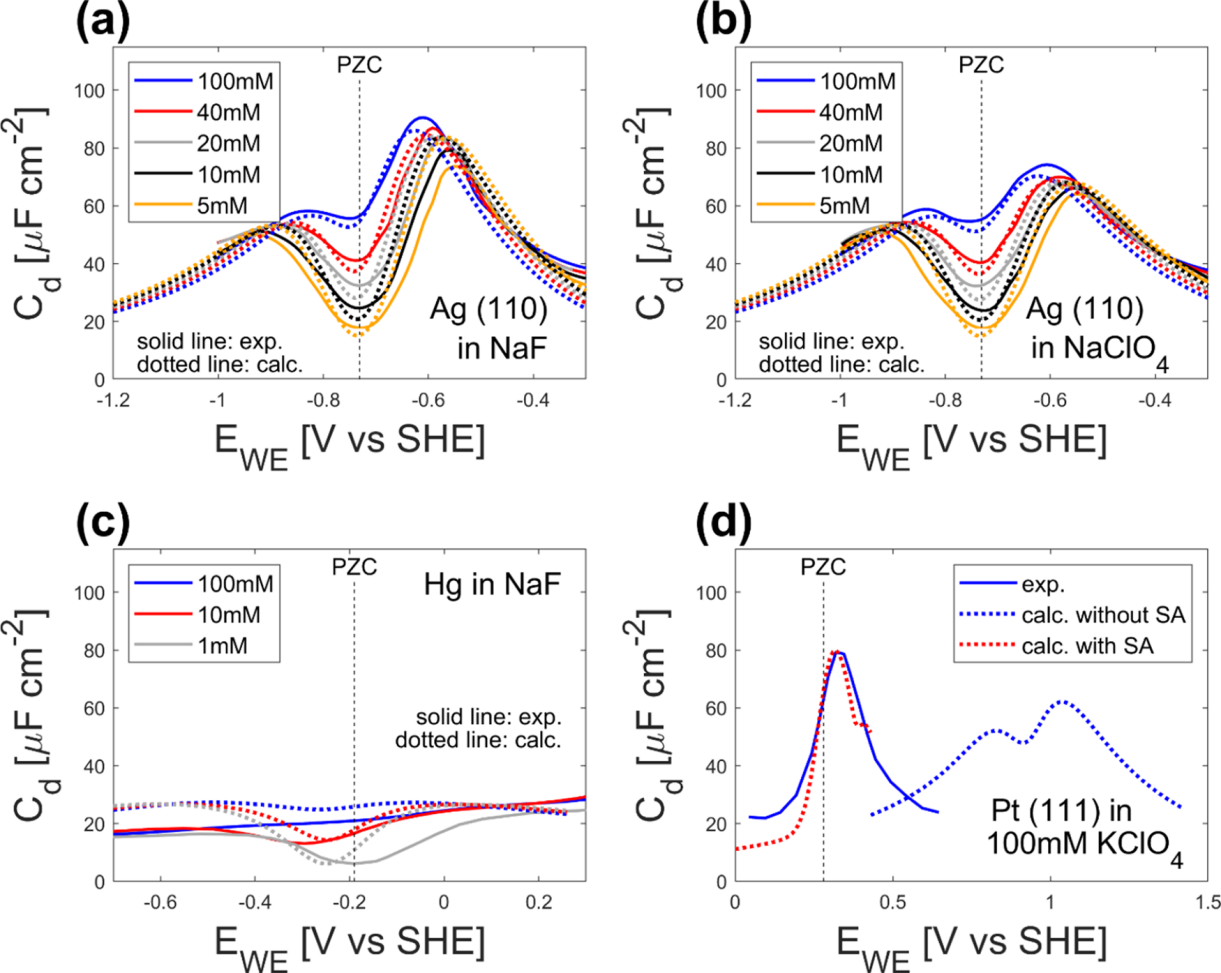
Comparison between the calculated (dotted lines) and experimental (solid lines) differential capacitance; (a) Ag(110) in NaF, (b) Ag(110) in NaClO_4_, (c) Hg in NaF, and (d) Pt(111) in 100 mM KClO_4_ (solid blue line: experimental, dotted blue line: calculated without specific interaction, dotted red line: calculated with specific interaction (eq 27)). Experimental results for (a, b) are extracted from ref (48), data for (c) are from ref (57), and data for (d) are from ref (4). The black dashed lines indicate the potential of zero charge (PZC).

At first, we analyzed Ag(110) in NaF and NaClO_4_ electrolytes and compared the differential capacitance predicted by the model with the experimental results by Valette,^48^ whose analysis suggests the effect of specific ion adsorption in this system is not significant. Figure 3a,b shows that our model captures the trends in the experiments: the differential capacitance has two local maxima and one local minimum. The local minimum increases when the ion concentration increases, and the height of the first local maximum (−0.9 to −0.8 V vs SHE), in the potential range below the PZC, is almost the same for NaClO_4_ and NaF, while the second local maximum (−0.65 to −0.55 V vs SHE), above the PZC, depends strongly on the anion species identity. Because the first local maximum can be attributed to cation adsorption (when the metal is negatively charged) and the second one is due to anion adsorption (when the metal is positively charged; see Section 3.5.), the change in the anion species affects only the second local maximum. The calculated height of the maxima and minima also agrees well with the experiment. Overall, the calculated results demonstrate good agreement with the experimental ones for all the electrolyte concentration and species without parameter fitting, suggesting the predictivity of the model.

Next, we checked the applicability of the model to different electrodes. As a system without significant specific adsorption, we calculated the differential capacitance on Hg in NaF solutions and compared it with the experimental results by Grahame,^57^ whose analysis suggests the specific ion adsorption is not significant in this system. The comparison between calculation and experimental results is shown in Figure 3c. Although the agreement is not perfect, the calculation successfully reproduces the different features of Hg compared to Ag: lower differential capacitance with significantly suppressed capacitance maxima. Considering that the model does not use any adjustable parameters, the agreement is satisfactory. The quantitative discrepancy between the experiment and calculation for Hg can probably be attributed to water chemisorption on Hg,^58^ which is not accounted for in the present model.

**Figure 4:**
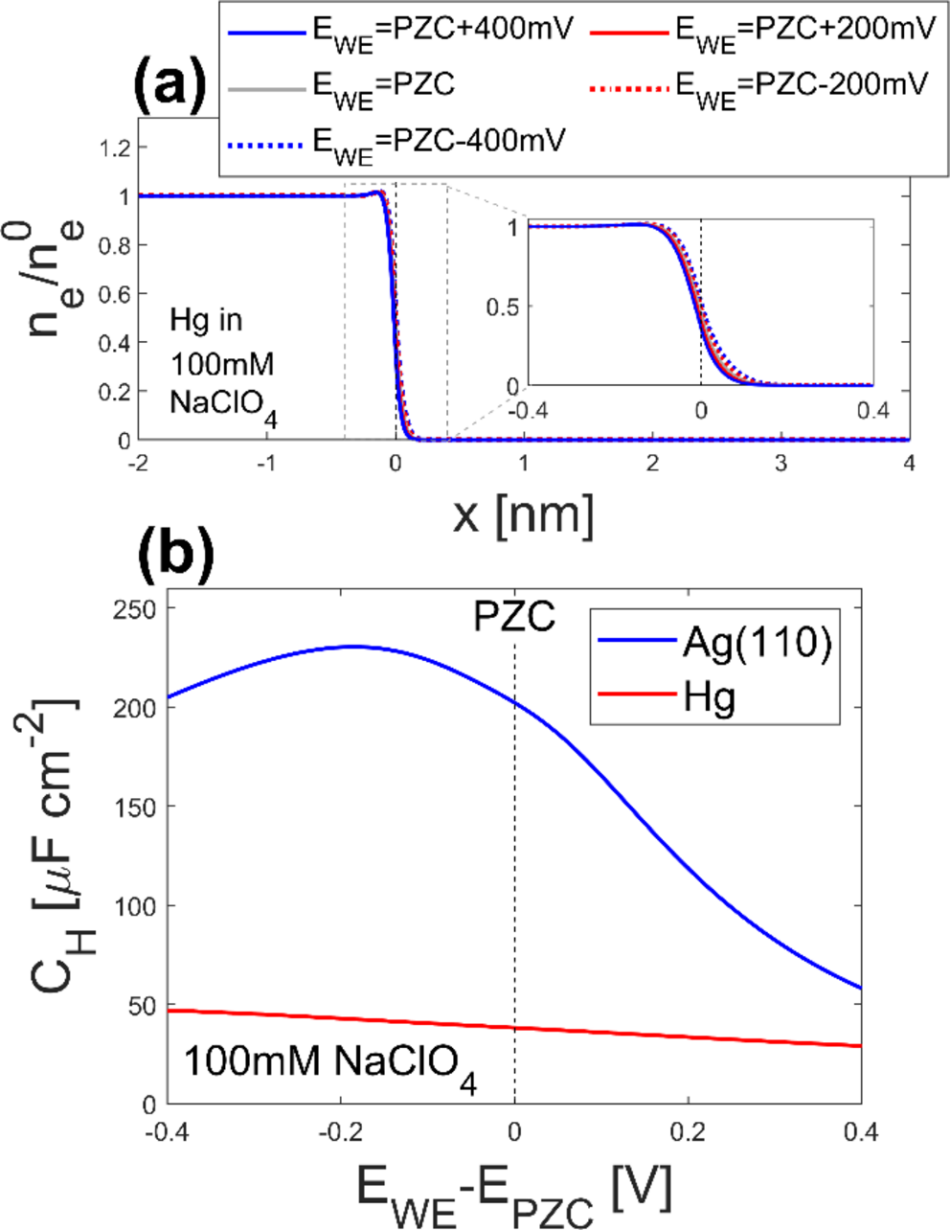
Calculated (a) electron density distributions on Hg in 100 mM NaClO_4_ and (b) Helmholtz capacitance (eq 26) of Hg (blue) and Ag(110) (red) electrodes in 100 mM NaClO_4_. For (a), the axis and line styles are the same as those in Figure 1b. The inset in (a) is the enlarged view of the domain inside the gray dashed box. The black dashed lines in (a, b) indicate the electrode–electrolyte interface and potential of zero charge (PZC), respectively.

The difference in *C*_d_ between Ag(110) and Hg can be attributed to the difference in the stability of the electrons in the metals. In our model, the metal bulk modulus is associated with the pseudopotential of electrons in the metal. Here, the bulk modulus of Hg is 0.267 Mbar,^50^ while that of Ag is 1.01 Mbar.^33^ As described in eq S29 in the SI, a larger bulk modulus results in higher pseudopotential in the metal because of stronger repulsive interactions from the core electrons. With higher pseudopotential, the electrons become less stable in the metal, and the electron density profile (*n*_e_(*x*)) becomes more sensitive to the applied electrode potential (compare Figures 1b and 4a). The larger sensitivity of *n*_e_(*x*) on *E*_WE_ results in a larger dσ_metal_/d*E*_WE_, which equals *C*_d_ (eq 25). When we focus on the Helmholtz capacitance (*C*_H_), the difference between the two electrodes becomes clearer as shown in Figure 4b. Here, *C*_H_ is calculated by
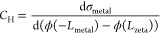
26where *L*_zeta_ = min(*r*_a_,*r*_c_) is the closest approach of ions.

Also, we compared the calculated differential capacitance on Pt(111) in 100 mM KClO_4_ with the experimental result by Pajkossy and Kolb^4^ to check the model applicability to a system with specific adsorption.^59,60^Figure 3d shows the results for the Pt(111) electrode. The figure shows a significant discrepancy between the experiment (the blue solid line) and the calculation (the blue dotted line) in terms of peak height, location, and number of peaks. The discrepancy is thought to be due to the specific interaction between Pt(111) surface and ClO_4_^–^ ion,^59^ which can be corrected by using an appropriate function for chemical potential for wall-anion interaction (μ_ws,a_) (see the red dotted line in Figure 3d). Here we used the form of Morse potential as
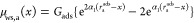
27where *G*_ads_ is the adsorption free energy (0.65 eV), α_1_ is the relaxation parameter (4.72 nm^–1^), and *r*_a_^ads^ is the effective radius of adsorbed anions (0.4 nm). Also, we accounted for the anione effective radius shift as *r*_a_(*x*) *= r*_a_^0^ + (*r*_a_^ads^ – *r*_a_^0^)Θ(*x* – 0.5(*r*_a_^0^+*r*_a_^ads^)), where *r*_a_^0^ is the effective anion radius in the bulk solution. Because of the lack of quantum-mechanical analysis (e.g., DFT calculation) on this interaction, we cannot conclude that the assumed interaction is physically reasonable. However, this result confirmed that the ion-electrode-specific interaction can significantly alter the differential capacitance and needs to be accounted for when applying the model to a system with specific adsorption. Also, it should be noted that quantum-mechanical analysis can be used to assess the significance of the specific interaction term in a certain system in case sufficient experimental data is unavailable. By running a DFT simulation with various ion-metal surface distances, one can obtain a free energy profile. This profile can serve as the wall-solute interaction term (μ_ws,a_(*x*)) in a similar manner to eq 27. If the adsorption free energy is sufficiently large, it will affect the calculated differential capacitance, which suggests the importance of including a specific interaction term in the model.

It is known that water molecules form a two-dimensional (2D) hydrogen bonding network on electrode surface like platinum^15^ that stabilize the water molecules and change their dielectric properties.^11−13^ Although our model includes the polarization energy that accounts for the stabilization of solvent dipole for the electric field, it does not include the explicit expressions for the two-dimensional hydrogen bonding in the vicinity of the interface. The model will be capable of including these effects by setting the parameters based on further analyses. The former effect, stabilization of water, prevents solute molecules from approaching the surface. This blocking effect can be included by obtaining the wall-solute interaction energy (μ_ws_) from DFT calculations with explicit water molecules, which evaluates the energy to put the solute molecule near the interface, including the reorganization energy of the water hydrogen bond. Also, the latter effect, a change in the dielectric properties, can be implemented by making the dielectric parameters (*e*.*g*., ϵ_s_^0^ and *a*_pol_) position-dependent. However, this analysis is beyond the scope of the current study and requires parameter fitting that is not readily available and/or is computationally cost-prohibitive.

### 3.3 Effect of Grand Potential Components

The effect of each grand potential component is analyzed by enabling them one by one in the calculation for Ag(110) in 100 mM NaF by substituting the total local grand potential (ω_tot_) in eq 5 with ω_tot_^(*k*)^ in Table 3.

**Table 3:** Expressions for ω_tot_^(*k*)^ to Analyze the Effects of the Major Potential Components^a^

expression for ω_tot_^(*k*)^	*r*_a_ [nm]	*r*_c_ [nm]	*a*_pol_ [nm/V]
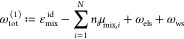	0.65	0.65	→0
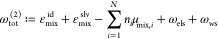	0.65	0.65	→0
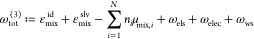	0.65	0.65	→0
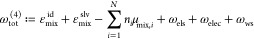	0.65	0.65	6
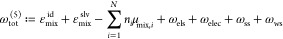	0.65	0.65	6
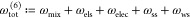	0.40	0.65	6

a For *k* = 1 and 2, the electron density is set as *n*_e_(*x*) = *n*_e_^0^Θ(−*x*).

The initial model (ω_tot_^(1)^) corresponds to the standard GCS theory. It accounts for the ideal mixing entropy (ε_mix_^id^), electrostatic energy (ω_els_), and wall-solute interaction (ω_ws_). Since the electron energy (ω_elec_) is missing in this model, the partial derivative of ω_tot_ with *n*_e_ and ∇*n*_e_ in eq 5 does not provide any meaningful information. Hence, instead of solving for the differential equation for *n*_e_, we manually set *n*_e_(*x*) = *n*_e_^0^Θ(−*x*), which results in neglecting the effect of the surface dipole due to electron spillover. This model also neglects the dielectric saturation due to the polarization of the solvent and solutes by setting *a*_pol_ → 0. In addition, the solute molecules are assumed to have the same size to exclude the effect of ion size differences. The second model (ω_tot_^(2)^) corresponds to the Bikerman model,^19^ which accounts for the effects of finite size of solute ions on mixing entropy (ε_mix_^slv^) by assuming all ions have the same size. The third model (ω_tot_^(3)^) adds the electron energy term (ω_elec_) to the second model that enables the evaluation of *n*_e_(*x*), which corresponds to the density potential functional theory developed by Huang.^20^ The fourth model (ω_tot_^(4)^) activates the dielectric saturation by setting *a*_pol_ = 6 nm/V, a fitted value to an *ab initio* simulation result.^17^ The fifth model (ω_tot_^(5)^) enables the solute–solute interaction (ω_ss_) to visualize its effect on the calculation results. Finally, the sixth model (ω_tot_^(6)^) includes the effect of ion size difference between anions and cations on the mixing entropy by activating ε_mix_^size^ and setting *r*_a_ ≠ *r*_c_.

**Figure 5:**
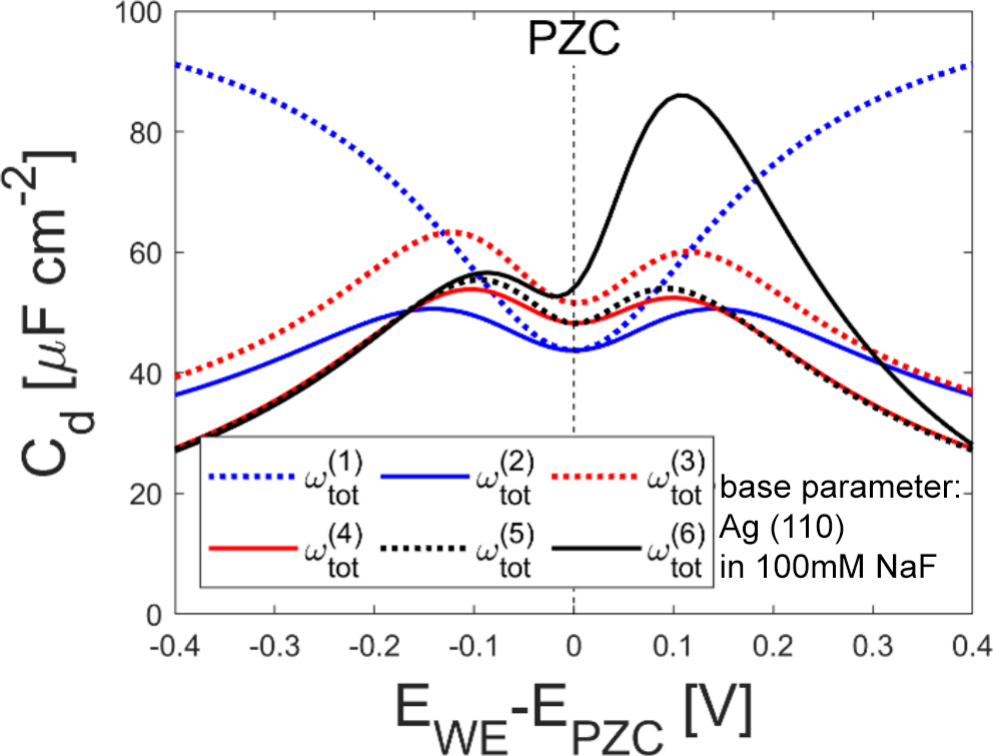
Comparison of calculated differential capacitance with ω_tot_^(k)^: The different line styles represent different expressions for ω_tot_ as shown in the legend. The parameter set for Ag(110) in 100 mM NaF was used as the base parameter.

Figure 5 shows that ω_tot_^(1)^ gives the differential capacitance with one local minimum, which saturates when the potential is far from the PZC as expected from the GCS theory. The line for ω_tot_^(2)^ shows that the addition of ε_mix_^slv^ results in the two local maxima, suggesting that they are due to the ions’ finite size. ε_mix_^slv^ reduces the capacitance far from the PZC because the limited availability of the space for solvent near the interface mitigates further ion accumulation. The line for ω_tot_^(3)^ shows that the electron energy (ω_elec_) increases the capacitance around PZC, and the effect is more significant in negative potential versus PZC than in positive potential. This is because the electron spillover becomes more significant in negative potential, where the electron is relatively unstable in the electrode (thus the Helmholtz capacitance *C*_H_ becomes larger in negative potential as shown in Figure 4b); this result is consistent with the discussion by Huang.^20^ The polarization of solvent and solute ions (*a*_pol_ ≠ 0) reduces the differential capacitance in the potential far from the PZC (the line for ω_tot_^(4)^). This is because the polarization of the ions and solvents induces dielectric saturation, which reduces the effective dielectric constant near the interface and thus the Stern layer capacitance when the electric field is strong.^16^ The addition of solute–solute interaction (ω_ss_) slightly increases the height of the peak (the line for ω_tot_^(5)^) because of the favorable interactions between the ions at higher ion strength. Although the effect of this interaction is not significant in case of monovalent electrolyte, it becomes larger as the ionic valence increases (see Figure S1 in the SI). The line for ω_tot_^(6)^ shows increased differential capacitance especially above the PZC. This is because the smaller size of the anion enhances the anion accumulation near the interface. Another analysis of the effects of the interaction terms, which uses a different order, also demonstrate significant effects of polarization of solvent and solute ions, as well as the size-dependent entropy (see Figure S2 in the SI). This analysis suggests that all the interactions introduced in this study, polarization of solvent and solute ions, size-dependent entropy, and solute–solute interactions, are responsible for predicting differential capacitance.

### 3.4 Parameter Sensitivity

**Figure 6:**
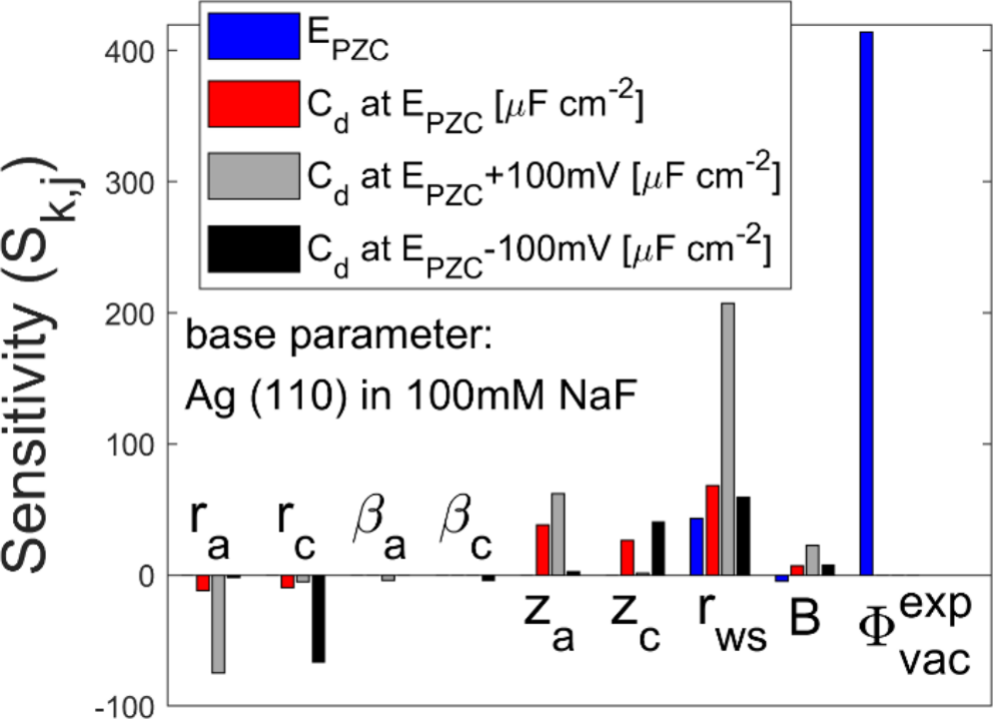
Sensitivity (eq 28) of EDL properties on the material properties. The different colors represent different material properties, as listed in the legend. The parameter set for Ag(110) in 100 mM NaF is used as the base parameters. *E*_PZ_ is in the unit of mV.

Parameter sensitivity analysis is conducted with the model to identify the critical material properties that impact the EDL structure. The parameters to be analyzed are the material properties introduced in Section 2.5: the effective hydrated radius of anion and cation (*r*_a_ and *r*_c_), polarizability of the hydrated shell of anion and cation (β_a_ and β_c_), ionic valence of anion and cation (*z*_a_ and *z*_c_), Wigner–Seitz radius of the electrode (*r_ws_*), bulk modulus of the electrode (*B*), and vacuum work function of the electrode (Φ_vac_). By changing these material properties by ±5% independently from the base parameters (values on Ag(110) in 100 mM NaF), we evaluated 4 properties of EDL structures: PZC (denoted as*E*_PZC_), *C*_d_ at *E*_PZC_, *C*_d_ at *E*_PZC_–0.1 V, and *C*_d_ at *E*_PZC_ + 0.1 V. The difference of ±5% is chosen as a value small enough to obtain a numerical derivative and large enough to neglect the effect of the numerical error due to solver tolerance. Then, the sensitivity of EDL property *k* on the material property *j* (*S*_*k*,*j*_) was calculated by
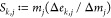
28where *m*_*j*_ is the base value of the material property *j*, Δ*e*_*k*,*j*_ is the shift of the EDL property *k* due to the shift in the material property *j*, and Δ*m*_*j*_ is the shift in the material property *j*. The calculated sensitivities of the EDL properties on the material properties are shown in Figure 6.

First, the effects of the electrolyte properties, *r*_a_, *r*_c_, β_a_, β_c_, *z*_a_, and *z*_c_ are discussed. Figure 6 demonstrates that none of these parameters have a recognizable effect on PZC, which confirms that PZC is independent of electrolyte properties in this model with no specific interactions. The properties of the anion (*r*_a_, β_a_, and *z*_a_) mainly affect *C*_d_ (*E*_PZC_ + 0.1 V), while the properties of the cation (*r*_c_, β_c_, and *z*_c_) show similar effects but in *C*_d_ (*E*_PZC_–0.1 V). Above the PZC, the anion accumulates near the interface (Figure 1c). Since the anion properties affect the anion’s accumulation affinity, they can impact the surface charge profile and thus the differential capacitance in high potential. The cation, on the other hand, accumulates under the PZC (Figure 1d) and thus the cation properties have stronger effects on the differential capacitance in the negative potential. The calculation results show that the electrolyte properties: *r*_a_, *r*_c_, *z*_a_, and *z*_c_ have large effects on the differential capacitance. Hence, one must take care of the effective ion radius and ionic valence to control the EDL structures.

In terms of the sensitivity of the electrode properties: *r*_ws_, *B*, and Φ_vac_, Figure 6 demonstrates that *r*_ws_ has a strong effect on all EDL properties. This strong effect can be attributed to the third-order effect of *r*_ws_ on *n*_e_^0^ (*n*_e_^0^:= 3/(4π*r*_ws_^3^)) and the impact of *n*_e_^0^ on the electron spillover (see the shift from ω_tot_^(2)^ to ω_tot_^(3)^ in Figure 5). Φ_vac_ significantly changes the PZC but shows no recognizable effects on differential capacitance. In this model, Φ_vac_ is used to evaluate Δϕ_WF_, which is a constant that simply shifts the absolute potential of the electrode. The shift in the absolute potential directly changes the PZC but does not change the shape of the potential-dependent differential capacitance. The bulk modulus (*B*) affects all of the EDL properties, but the impact is smaller than *r*_ws_. Even with the small sensitivity, it was the main reason for the difference between Ag(110) and Hg, as discussed in Section 3.2, because of the large difference in the bulk modulus (1.01 Mbar for silver^33^ and 0.267 Mbar for mercury^50^). The positive sensitivity of *C*_d_ on *B* is consistent with the smaller *C*_d_ on Hg than that on Ag(110). Although the Wigner–Seitz radius is the most sensitive property of the electrode, one needs to also consider the bulk modulus as an important property to predict the differential capacitance.

### 3.5 Potential Applications of the Model

Since this model provides a means to predict the structure of EDLs without parameter fitting, it can be applied in various applications, including different materials and reaction microenvironments, whereas conventional continuum EDL models require parameter fitting based on the experimental results. For example, Huang’s model^20^ calibrates the optical dielectric constant based on the experimental differential capacitance. Since the model presented herein does not require parameter fitting, it can be used to predict the EDL structure without experimental data, which gives us insights into material selection to control the surface properties.

Also, the continuum descriptions developed in this study can be extended into higher dimensions to address the inhomogeneous nature of the electrode surface, which plays a significant role in electrocatalytic processes.^15^ To fully account for the inhomogeneity of the interface, the model can be extended into three-dimensional (3D) space and then coupled with quantum-mechanical simulations by incorporating the present model as an implicit solvent model in DFT simulations.^27,61−64^ The present model would improve the accuracy of DFT simulations by providing a more accurate description of the interactions in the electrolyte. Although our model might increase the computational cost due to the need for iterative calculations for solute molecule distributions (*n*_*i*_), it would improve the calculation accuracy of the implicit water model because of the improved descriptions in the size-dependent entropy term and the polarization model. One should note that in this application, the calculation becomes three-dimensional so that we no longer need to include the solute–solute interaction (ω_ss_), which is derived by integrating the Coulomb interaction in the *y*–*z* direction as a special treatment for a one-dimensional model.

## 4 Conclusions

In this study, a predictive multiscale continuum model was developed for the electric double layer that eliminates the need for parameter fitting due to the incorporation of microscopic interactions and independent material properties. The model reproduced the trends in the experimental differential capacitance with multiple noninteracting electrode and electrolyte materials (Ag(110) in NaF, Ag(110) in NaClO_4_, and Hg in NaF), which verifies the accuracy and predictiveness of the model. However, the poorer predictions for Pt(111) in KClO_4_ suggest the necessity of further study to capture the effect of electrode-ion-specific interactions in a parameter-fitting-free manner. The difference in the differential capacitance between Hg and Ag(110) was attributed to the difference in the electron stability in the metal. Sensitivity analyses confirmed that all the newly incorporated interactions added in this study play a role in predicting the differential capacitance. It also demonstrated the effective ion radius, the ionic valence, the electrode’s Wigner–Seitz radius, and the bulk modulus of the electrode are significant material properties that control the EDL structure. The model framework and findings provide insights into the EDL structures and enable predictive evalumation of EDLs with low computational cost.

## Supporting Information Available

List of equations and parameters (Tables S1–S3), derivation of the equations, PDE expressions, and additional calculation results (Figures S1 and S2) (PDF)
