# Supplementary material for: Parameter-Fitting-Free Continuum Modeling of Electric Double Layer in Aqueous Electrolyte
Source: J Chem Theory Comput. 2024 Jul 5;20(14):6184–96. doi: 10.1021/acs.jctc.4c00408 (PMC11270741; doi:10.1021/acs.jctc.4c00408)
Supplement: Supplementary file 1 — ct4c00408_si_001.pdf [file ct4c00408_si_001.pdf]

Supporting Information for

Parameter-fitting-free Continuum Modeling for Electric Double Layer in Aqueous Electrolyte

Masao Suzuki Shibata [a,b,*] , *Yu Morimoto* [a], *Iryna V. Zenyuk* [a], *Adam Z. Weber* [b]

a: Department of Chemical and Biomolecular Engineering; National Fuel Cell Research Center, University of California, Irvine, Irvine, CA 92697.

b: Energy Conversion Group, Lawrence Berkeley National Laboratory, 1 Cyclotron Rd, Berkeley, CA 94720.

*: masaos@lbl.gov

Table of Contents

S1. List of equations and parameters 2
S2. Derivation of the equations 4
S2.1. Mixing Entropy 4
S2.2. Solute-Solute interaction 6
S2.3. Derivation of pseudopotential from the bulk modulus 8
S3. PDE expressions 9
S3.1. PDE for electrostatic potential 9
S3.2. PDE for electron energy 9
S3.3. PDEs for species density 9
S4. Additional calculation results 10
S4.1. Effect of solute-solute interactions in multivalent electrolytes 10
S4.2. Effect of grand potential components in different order from Section 3.3. 11
References 12

S1. List of equations and parameters

Table S1. Equations for local grand potentials

	EQUATIONS	REF
$\boldsymbol{\omega_{tot}}$	$\omega_{tot}(\phi, \nabla\phi, n_e, \nabla n_e, n_a, n_c, x) \coloneqq \omega_{mix}(n_a, n_c) + \omega_{els}(\phi, \nabla\phi, n_e, n_i, x) + \omega_{elec}(n_e, \nabla n_e, x) + \omega_{ss}(n_a, n_c) + \omega_{ws}(n_a, n_c, x)$	Main 2.1
$\boldsymbol{\omega_{mix}}$	$\omega_{mix}(n_a, n_c) \coloneqq \varepsilon_{mix}^{id}(n_a, n_c) + \varepsilon_{mix}^{slv}(n_a, n_c) + \varepsilon_{mix}^{size}(n_a, n_c) - \sum_{i=a,c} n_i \mu_{mix,i}$	SI S2.1
	$\varepsilon_{mix}^{id}(n_a, n_c) \coloneqq k_B T(n_c \ln n_c V_c + n_a \ln n_a V_a)$	SI S2.1
	$\varepsilon_{mix}^{slv}(n_a, n_c) \coloneqq k_B T \frac{(1-n_a V_a - n_c V_c)}{V_a} \ln(1 - n_a V_a - n_c V_c)$	SI S2.1
	$\varepsilon_{mix}^{size}(n_a, n_c) \coloneqq k_B T \left(\frac{1}{V_c} - \frac{1}{V_a}\right)(1 - n_c V_c)\ln(1 - n_c V_c)$	SI S2.1
	$\mu_{mix,i} \coloneqq \frac{d}{dn_i^0}\left(\varepsilon_{mix}^{id}(n_a^0, n_c^0) + \varepsilon_{mix}^{slv}(n_a^0, n_c^0) + \varepsilon_{mix}^{size}(n_a^0, n_c^0)\right)$ (for $i = a, c$)	SI S2.1
$\boldsymbol{\omega_{els}}$	$\omega_{els}(\phi, \nabla\phi, n_e, n_i, x) \coloneqq \omega_{els}^{ef}(\nabla\phi, x) + \omega_{els}^{chg}(\phi, n_e, n_a, n_c, x) + \omega_{els}^{pol}(\nabla\phi, n_a, n_c, x)$	Main 2.3.2
	$\omega_{els}^{ef}(\nabla\phi, x) \coloneqq -\frac{1}{2}\epsilon_0 \left(\epsilon_{op}^m \Theta(-x) + \epsilon_{op}^s \Theta(x)\right)\lvert\nabla\phi\rvert^2$	Ref [1, 2]
	$\omega_{els}^{chg}(\phi, n_e, n_a, n_c, x) \coloneqq e_0 \phi(n_e^0 \Theta(-x) - n_e + z_a n_a + z_c n_c)$	Ref [1, 2]
	$\omega_{els}^{pol}(\nabla\phi, n_a, n_c, x) \coloneqq -\frac{3\epsilon_0\left(\epsilon_{eff}^{\nabla\phi=0}(n_a, n_c) - \epsilon_{op}^s\right)}{a_{pol}^2}\Theta(x)\ln\left(\frac{\sinh(a_{pol}\nabla\phi)}{a_{pol}\nabla\phi}\right)$	Main 2.3.2
	$\epsilon_{eff}^{\nabla\phi=0}(n_a, n_c) \coloneqq \epsilon_s^0 - (n_a\beta_a + n_c\beta_c)/N_{avo}$	Main 2.3.2
$\boldsymbol{\omega_{elec}}$	$\omega_{elec}(n_e, \nabla n_e, x) \coloneqq \varepsilon_{elec}^{txc}(n_e, \nabla n_e) + \varepsilon_{elec}^{ps}(n_e, x) - n_e \mu_{elec}$	Main 2.3.3
	$\varepsilon_{elec}^{txc}(n_e, \nabla n_e) \coloneqq \frac{e_{au}}{a_0^3}\left[\frac{3}{10}(3\pi^2)^{\frac{2}{3}} a_0^5 n_e^{\frac{5}{3}} - \frac{3}{4}\left(\frac{3}{\pi}\right)^{\frac{1}{3}} a_0^4 n_e^{\frac{4}{3}} - \frac{0.056 a_0^4 n_e^{\frac{4}{3}}}{0.079 + a_0 n_e^{\frac{1}{3}}} + \frac{a_0^5}{72}\frac{(\nabla n_e)^2}{n_e}\right]$	Ref [3]
	$\varepsilon_{elec}^{ps}(n_e, x) \coloneqq n_e\left(\mu_{ps}^m \Theta(-x) + \mu_{ps}^s \Theta(x)\right)$	Main 2.3.3
	$\mu_{elec} \coloneqq -e_0(E_{WE,abs} - \Delta\phi_{WF})$	Main 2.3.3
$\boldsymbol{\omega_{ss}}$	$\omega_{ss}(n_a, n_c) \coloneqq \varepsilon_{ss}^{ion}(n_a, n_c) - (z_a^2 n_a + z_c^2 n_c)\mu_{ss}^{ref}$	SI S2.2
	$\varepsilon_{ss}^{ion}(n_a, n_c) \coloneqq -\frac{k_B T}{4\pi r_{ave}^3}\left[\ln\left(\frac{\pi}{2} j x_{ss} Y_1\left(j x_{ss}(n_a, n_c)\right)\right) + \frac{1}{2}\left(x_{ss}(n_a, n_c)\right)^2\right]$	SI S2.2
	$\mu_{ss}^{ref} \coloneqq \frac{e_0^2}{8\pi\epsilon_0\epsilon_{eff}^0 r_{ave}}\left(\frac{real\left(Y_0\left(jix_{ss}(n_a^0, n_c^0)\right)\right)}{real\left(jix_{ss}(n_a^0, n_c^0) Y_1\left(jix_{ss}(n_a^0, n_c^0)\right)\right)} - 1\right)$	SI S2.2
$\boldsymbol{\omega_{ws}}$	$\omega_{ws}(n_a, n_c, x) = n_a \mu_{ws,a}(x) + n_c \mu_{ws,c}(x)$	Main 2.3.5
	$\mu_{ws,a}(x) \coloneqq \mu_{cut}\Theta(r_a - x)$	Main 2.3.5
	$\mu_{ws,c}(x) \coloneqq \mu_{cut}\Theta(r_c - x)$	Main 2.3.5

'Main' and 'SI' represent sections in the main text and this supporting information, respectively. $\Theta(x)$ is the Heaviside step function which takes 1 when x>0 and 0 when x<0.

Table S2. Equations for dependent variables

NAME	EQUATIONS	DESCRIPTION
n_e^0	$3/(4\pi r_{ws}^3)$	Electron density in bulk metal
n_i^0	$c_i^0 N_{avo}$ (for $i = a, c$)	Number density of solutes in the bulk
V_i	$4\pi r_i^3/3$ (for $i = a, c$)	Molecular volume of ions
μ_{ps}^m	Eq. S29	Electron pseudopotential in metal (SI 2.3)
μ_{ps}^s	-0.043 eV	Electron pseudopotential in solution
r_{ave}	$\frac{1}{2}(r_a + r_c)$	Average ion radius
ϵ_{eff}^0	$\epsilon_s^0 - \sum_{i=1}^N (n_i^0 \beta_i / N_{avo})$	Effective dielectric constant in the bulk
$\epsilon_{eff}^{\nabla\phi=0}$	$\epsilon_s^0 - \sum_{i=1}^N (n_i \beta_i / N_{avo})$	Effective dielectric constant without electric field
ϵ_{eff}	Eq. S32	Effective dielectric constant
l_D	$\left[k_B T \epsilon_0 \epsilon_{eff}^0 / (e_0^2 \sum_{i=1}^N z_i^2 n_i^0)\right]^{0.5}$	Debye length
L_{sol}	$\max(20 l_D, 6\text{ nm})$	Length of the solution domain
x_{ss}	$\left[e_0^2 r_{ave}^2 (\sum_{i=1}^N z_i^2 n_i) / (\epsilon_0 \epsilon_{eff}^0 k_B T)\right]^{0.5}$	Intermediate variable for ω_{ss}
$\Delta\phi_{WF}$	$\Phi_{vac}^{exp} - \Phi_{vac}^{calc}$, see Table 1	Potential correction for work function

Table S3. Constants.

NAME	VALUES	DESCRIPTION
k_B	1.381×10^{-23} J/K	Boltzmann constant
e_0	1.602×10^{-19} C	Elementary charge
ϵ_0	8.854×10^{-12} F/m	Vacuum permittivity
N_{avo}	6.022×10^{23} /mol	Avogadro number
a_0	5.292×10^{-11} m	Bohr radius
e_{au}	4.360×10^{-18} J	Hartree energy
T	298 K	Temperature of the system
ϵ_{op}^m	1.00	Optical dielectric constant in the metal phase
ϵ_{op}^s	1.76	Optical dielectric constant in the solution phase
ϵ_s^0	80.1	Dielectric constant of the bulk solvent
j	$\sqrt{-1}$	Imaginary unit
a_{pol}	6.0×10^{-9} m/V	Slope for dielectric saturation (Fit to Ref [4], SI 3.2)
μ_{cutoff}	1000 eV	Cutoff energy
L_{metal}	3 nm	Length of the metal domain

See Table 1 and 2 in the main text for values of material properties (r_{ws}, B, Φ_{vac}^{exp}, r_i, β_i, and z_i).

S2. Derivation of the equations

S2.1. Mixing Entropy

The Boltzmann equation relates the entropic energy (ω_{mix}) to the partition function (W) as:

$$\omega_{mix} \coloneqq -\frac{k_B T}{V} \ln \frac{W}{W_{ref}}, \tag{S1}$$

where V is the volume of the system of interest, W is the partition function, and W_{ref} is the partition function in the bulk solution. W is obtained from a lattice model for multiple species with different molar volumes. Here we only consider the partition function due to mixing. At first, let us consider the partition function to put the first species ($i = 1$). With a lattice model, the number of microstates equals to the number of cases to choose $n_1 V$ boxes from V/V_1 boxes.

$$W_1 = \frac{(V/V_1)!}{(n_1 V)!\,(V(1 - n_1 V_1)/V_1)!}, \tag{S2}$$

where the n_i is the species density (number per unit volume) and V_i is the volume of the species. For next species, the remaining space is reduced into $V(1 - n_1 V_1)$ and the partition function becomes:

$$W_2 = \frac{(V(1 - n_1 V_1)/V_2)!}{(n_2 V)!\,(V(1 - n_1 V_1 - n_2 V_2)/V_2)!}, \tag{S3}$$

where we neglected the steric interference and assumed all the remaining space is available to put $i = 2$ species. The same applies to $i \geq 3$ so that:

$$W_i = \frac{\left(V\left(1 - \sum_{j<i} n_j V_j\right)/V_i\right)!}{(n_i V)!\,\left(V\left(1 - \sum_{j\leq i} n_j V_j\right)/V_i\right)!}, \tag{S4}$$

The total partition function (W) becomes:

$$W = \prod_i W_i = \prod_i \left[\frac{\left(\frac{V}{V_i}\left(1 - \sum_{j<i} n_j V_j\right)\right)!}{(n_i V)!\left(\frac{V}{V_i}\left(1 - \sum_{j\leq i} n_j V_j\right)\right)!}\right], \tag{S5}$$

One should note that this loading process should start from the bigger species and move to smaller ones to minimize the error due to steric interference. By taking the logarithm,

$$\ln W = -\sum_i \left[\ln\{(n_i V)!\} + \ln\left\{\left(\frac{V}{V_i}\left(1 - \sum_{j\leq i} n_j V_j\right)\right)!\right\} - \ln\left\{\left(\frac{V}{V_i}\left(1 - \sum_{j<i} n_j V_j\right)\right)!\right\}\right], \tag{S6}$$

By assuming a large system (large V), the Stirling approximation ($\ln n! \approx n \ln n - n$) gives:

$$\frac{\ln W}{V} = -\sum_{i=1}^{N} [n_i \ln n_i V_i] - \frac{\eta_N}{V_N} \ln \eta_N - \sum_{i=1}^{N-1} \left[\left(\frac{1}{V_i} - \frac{1}{V_{i+1}} \right) \eta_i \ln \eta_i \right], \tag{S7}$$

where $\eta_i \coloneqq 1 - \sum_{k \le i} n_k V_k$ and N is the number of species. Thus, Eq. S1, can be expressed as

$$\omega_{mix} = \varepsilon_{mix}^{id} + \varepsilon_{mix}^{slv} + \varepsilon_{mix}^{size} - \sum_{i} n_i \mu_{mix,i}, \tag{S8}$$

where

$$\varepsilon_{mix}^{id} \coloneqq k_B T \sum_{i=1}^{N} n_i \ln n_i V_i, \tag{S9}$$

$$\varepsilon_{mix}^{slv} \coloneqq k_B T \frac{\eta_N}{V_N} \ln \eta_N, \tag{S10}$$

$$\varepsilon_{mix}^{size} \coloneqq k_B T \sum_{i=1}^{N-1} \left[\left(\frac{1}{V_i} - \frac{1}{V_{i+1}} \right) \eta_i \ln \eta_i \right], \tag{S11}$$

$$\mu_{mix,i} \coloneqq \frac{d}{dn_i} \left(\varepsilon_{mix}^{id} + \varepsilon_{mix}^{slv} + \varepsilon_{mix}^{size} \right) \Big|_{n_i = n_i^0}, \tag{S12}$$

Note that if all the species have the same volume ($V_i = V_0$), it becomes the Bikerman's entropy term [5]:

In the model developed in this study, $i = 1$ represents the cation and $i = 2$ represents the anion. We assume it is a strong electrolyte so that the ions are fully dissociated. We assume the solvent simply fills the space that is not occupied by the cation nor the anion ($n_s V_s = 1 - n_c V_c - n_a V_a$), which makes the partition function for solvent (W_3) unity. Hence, $N = 2$ and can be written as

$$\varepsilon_{mix}^{id} \coloneqq k_B T (n_a \ln n_a V_a + n_c \ln n_c V_c), \tag{S13}$$

$$\varepsilon_{mix}^{slv} = k_B T \frac{1 - n_a V_a - n_c V_c}{V_a} \ln(1 - n_a V_a - n_c V_c), \tag{S14}$$

$$\varepsilon_{mix}^{size} = k_B T \left(\frac{1}{V_c} - \frac{1}{V_a} \right) (1 - n_c V_c) \ln(1 - n_c V_c), \tag{S15}$$

S2.2. Solute-Solute interaction

Here we consider solute-solute electrostatic interaction that is not included in ω_{els}. ω_{els} accounts for the electrostatic in the perpendicular direction to the electrode surface (x direction). However, it assumes a homogeneous ion distribution in the plane parallel to the electrode ($y-z$ plane). In reality, ions can re-distribute in $y-z$ direction as well due to the electrostatic interaction between solute molecules. Near the interface, ionic strength can be large due to the ion accumulation and, thus, we include a correction term for the ion distribution in $y-z$ plane.

As a primary model, we use the same assumptions as the Debye-Hückel theory:

- Solvent and solute molecules other than the 'central ion' serve as a continuum medium with a certain dielectric constant, and closest approach to the central ion.
- The dielectric constant is constant in the solution.
- The control volume is thin enough to neglect the distribution in x direction.

We also assumed that all of the ions in the solution have the same effective radius (r_{ave}) in the derivation of solute-solute interaction energy for simplification. This assumption has a minor effect on calculated differential capacitance (root-mean-square error of 1.2 μF/cm^2 with parameters for Ag(110) in 100M NaF).With these assumptions, we employ Poisson-Boltzmann equation in a cylindrical coordinate with homogeneous distribution in the vertical direction:

$$\nabla^2\phi(r) = -\frac{e_0}{\epsilon_0\epsilon_{eff}^0}\sum_k z_k n_k \exp\left(-\frac{z_k e_0 \phi(r)}{k_B T}\right), \tag{S16}$$

where ϕ is the electrostatic potential, ϵ_0 is the vacuum permittivity, ϵ_{eff}^0 is the effective dielectric constant ($\epsilon_{eff}^0 \coloneqq \epsilon_s^0 - \sum_{i=1}^N (n_i^0\, \beta_i/N_{avo})$), z_k is ionic valency, e_0 is the elementary charge, and n_k is the average ion density in the control volume. The subscript k represents the solute species. Here we consider the electrostatic potential due to a central ion i located at $r=0$ in a cylindrical coordinate with homogeneous distribution in angular and height direction. By decomposing the Laplace operator (∇^2) in cylindrical coordinates, the equation becomes

$$\frac{d^2\phi}{dr^2} + \frac{1}{r}\frac{d\phi}{dr} = -\frac{e_0}{\epsilon_0\epsilon_{eff}^0}\sum_k z_k n_k \exp\left(-\frac{z_k e_0 \phi(r)}{k_B T}\right), \tag{S17}$$

By assuming $|z_k e_0 \phi/k_B T| \ll 1$, the first-order approximation of the exponent factor can be applied to obtain,

$$\frac{d^2\phi}{dr^2} + \frac{1}{r}\frac{d\phi}{dr} - \kappa_1^2\phi + \kappa_0^2\phi_0 = 0 \tag{S18}$$

where $\kappa_0 \coloneqq \sqrt{\frac{e_0^2}{\epsilon_0\epsilon_{eff}^0 k_B T}\sum_k z_k n_k}$, $\kappa_1 \coloneqq \sqrt{\frac{e_0^2}{\epsilon_0\epsilon_{eff}^0 k_B T}\sum_k z_k^2 n_k}$, and $\phi_0 \coloneqq \frac{k_B T}{e_0}$. The general solution for this differential equation is

$$\phi(r) = \frac{\kappa_0^2}{\kappa_1^2}\phi_0 + A_1 J_0(j\kappa_1 r) + A_2 \mathrm{real}\big(Y_0(j\kappa_1 r)\big) \tag{S19}$$

where $J_0$is the Bessel function of the first kind of order zero, Y_0 is the Bessel function of the second kind of order zero, and j is the unit imaginary unit. Here, the boundary conditions for potential around ion i to be satisfied are:

$$\phi(\infty) = \text{finite}, \qquad \left.\frac{d\phi}{dr}\right|_{r=r_{ave}} = -\frac{z_i e_0}{4\pi\epsilon_0\epsilon_{eff}^0 r_{ave}^2} \tag{S20}$$

Hence, we get, $A_1 = 0$ and $A_2 = \frac{z_i e_0}{4\pi\epsilon_0\epsilon_{eff}^0 r_{ave}}\left\{\mathrm{real}\big(jx_{ss}Y_1(jx_{ss})\big)\right\}^{-1}$, where Y_1 is the Bessel function of the second kind of order one, and $x_{ss} \coloneqq \kappa_1 r_{ave}$. Thus, we obtain

$$\phi(r) = \frac{\kappa_0^2}{\kappa_1^2}\phi_0 + \frac{z_i e_0}{4\pi\epsilon_0\epsilon_{eff}^0 r_{ave}}\frac{\mathrm{real}\big(Y_0(j\kappa_1 r)\big)}{\mathrm{real}\big(jx_{ss}Y_1(jx_{ss})\big)} \tag{S21}$$

Since the electrostatic potential due to the central ion is $\phi_{central}(r) = \frac{z_i e_0}{4\pi\epsilon_0\epsilon_{eff}^0 r}$, the electrostatic potential due to the other ions is defined as: $\phi_{other} \coloneqq \phi - \phi_{central}$. From this, the electrochemical potential due to the solute-solute interaction is:

$$\mu_{ss,i} = \frac{1}{2} z_i e_0\big(\phi_{other}(r_{ave}) - \phi_{other}(\infty)\big) = z_i^2 \mu_{ss} \tag{S22}$$

where $\mu_{ss} \coloneqq \frac{e_0^2}{8\pi\epsilon_0\epsilon_{eff}^0 r_{ave}}\left(\frac{\mathrm{real}(Y_0(jx_{ss}))}{\mathrm{real}(jx_{ss}Y_1(jx_{ss}))} - 1\right)$. The 1/2 sign in Eq. S22 is needed because the interaction between i and k are accounted for twice in $\mu_{ss,i}$ and $\mu_{ss,k}$. The energy ε_{ss} satisfies $\partial\varepsilon_{ss}/\partial n_i \coloneqq \mu_{ss,i}$ and $\varepsilon_{ss}(x_{ss} = 0) = 0$. Thus,

$$d\varepsilon_{ss} = \sum\nolimits_i \mu_{ss,i}\, dn_i = \frac{k_B T x_{ss}}{4\pi r_{ave}^3}\left(\frac{\mathrm{real}\big(Y_0(jx_{ss})\big)}{\mathrm{real}\big(jx_{ss}Y_1(jx_{ss})\big)} - 1\right) dx_{ss} \tag{S23}$$

Hence,

$$\begin{aligned} \varepsilon_{ss} = \int_0^{\varepsilon_{ss}} d\varepsilon_{ss} &= \frac{k_B T}{4\pi r_{ave}^3}\int_0^{x_{ss}} x_{ss}\left(\frac{\mathrm{real}\big(Y_0(jx_{ss})\big)}{\mathrm{real}\big(jx_{ss}Y_1(jx_{ss})\big)} - 1\right) dx_{ss} \\ &= -\frac{k_B T}{4\pi r_{ave}^3}\left[\ln\left(\frac{\pi}{2} jx_{ss}Y_1(jx_{ss})\right) + \frac{1}{2}x_{ss}^2\right] \end{aligned} \tag{S24}$$

S2.3. Derivation of pseudopotential from the bulk modulus

The electron pseudopotential in the metal, μ_{ps}^{m}, is calculated from the experimental bulk modulus, B. This section shows the derivation of the relationship between μ_{ps}^{m} and B. The bulk modulus is a fundamental property of a metal that is attributed to the energy shift due to a volume change and is calculated as

$$B = V\left(\frac{\partial^2 E}{\partial V^2}\right)_N, \tag{S25}$$

where V is the volume of the bulk metal, N is the number of electrons in the bulk metal ($N = n_e^0 V$), and E is the total electron energy of the bulk metal ($\varepsilon_0 N$). ε_0 is the energy per electron and is expressed as

$$\varepsilon_0 = \frac{\varepsilon_{elec}^{txc}(n_e^0, 0) + n_e^0 \mu_{ps}^{m}}{n_e^0} = \varepsilon_0' + \mu_{ps}^{m} \quad \left(\text{where } \varepsilon_0' \coloneqq \frac{\varepsilon_{elec}^{txc}(n_e^0, 0)}{n_e^0}\right). \tag{S26}$$

Using the structureless expression by Perdew et al. [6], the pseudopotential μ_{ps}^{m} is expressed as

$$\mu_{ps}^{m} = 2\pi e_{au} a_0 n_e^0 r_{core}^2 - \frac{3}{10} e_{au} a_0 z^{\frac{2}{3}} \left(\frac{4\pi}{3}\right)^{\frac{1}{3}} (n_e^0)^{\frac{1}{3}}, \tag{S27}$$

where the first term in the right side represents repulsive interaction from the core electron in the Ashcroft pseudopotential [7] and the second term stems from the summation of Madelung energy and the electrostatic self-energy. r_{core} is the core radius and is a parameter that is determined from the bulk modulus. From these equations, one can obtain,

$$r_{core}^2 = \frac{1}{4\pi e_{au} a_0}\left[B(n_e^0)^{-2} - 2\frac{\partial \varepsilon_0'}{\partial n_e^0} - n_e^0 \frac{\partial^2 \varepsilon_0'}{\partial (n_e^0)^2} + \frac{2}{15} e_{au} a_0 z^{\frac{2}{3}} \left(\frac{4\pi}{3}\right)^{\frac{1}{3}} (n_e^0)^{-\frac{2}{3}}\right], \tag{S28}$$

Hence,

$$\mu_{ps}^{m} = \frac{B}{2n_e^0} - n_e^0 \frac{\partial \varepsilon_0'}{\partial n_e^0} - \frac{1}{2}(n_e^0)^2 \frac{\partial^2 \varepsilon_0'}{\partial (n_e^0)^2} - \frac{7}{30} e_{au} a_0 z^{\frac{2}{3}} \left(\frac{4\pi}{3}\right)^{\frac{1}{3}} (n_e^0)^{\frac{1}{3}}, \tag{S29}$$

In terms of $\psi_e^0 = (n_e^0)^{\frac{1}{3}}$, μ_{ps}^{m} can also be expressed as

$$\mu_{ps}^{m} = \frac{B}{2(\psi_e^0)^3} - \frac{2}{9}\psi_e^0 \frac{\partial \varepsilon_0'}{\partial \psi_e^0} - \frac{1}{18}\frac{\partial^2 \varepsilon_0'}{\partial {\psi_e^0}^2}(\psi_e^0)^2 - \frac{7}{30} e_{au} a_0 z^{\frac{2}{3}} \left(\frac{4\pi}{3}\right)^{\frac{1}{3}} \psi_e^0, \tag{S30}$$

S3. PDE expressions

The distributions of the electrostatic potential (ϕ), electron density (n_e), and species density (n_i) are obtained by minimizing the total grand potential as described in the main text. The condition to minimize the total grand potential gives three partial differential equations (PDEs) as shown in Eq. 5. In the actual calculation, these PDEs are modified in a way that stabilizes the calculation. Here we describe how we formulated the PDEs.

S3.1. PDE for electrostatic potential

The equation for ϕ: $\frac{\partial \omega_{tot}}{\partial \phi} - \nabla\left(\frac{\partial \omega_{tot}}{\partial \nabla \phi}\right) = 0$, is modified to a form of Poisson equation as

$$\nabla\left(\epsilon_0 \epsilon_{eff} \nabla \phi\right) = -e_0\left(n_e^0 \Theta(-x) - n_e + z_a n_a + z_c n_c\right), \tag{S31}$$

where

$$\epsilon_{eff} = \epsilon_{op}^m \Theta(-x) + \left\{\epsilon_{op}^s + 3\left(\epsilon_{eff}^{\nabla\phi=0} - \epsilon_{op}^s\right)\left(\frac{\coth\left(a_{pol}\nabla\phi\right)}{a_{pol}\nabla\phi} - \frac{1}{\left(a_{pol}\nabla\phi\right)^2}\right)\right\}\Theta(x), \tag{S32}$$

S3.2. PDE for electron energy

The equation for n_e: $\frac{\partial \omega_{tot}}{\partial n_e} - \nabla\left(\frac{\partial \omega_{tot}}{\partial \nabla n_e}\right) = 0$, is modified by using an intermediate variable $\psi_e \coloneqq n_e^{1/3}$ as

$$\nabla^2 \psi_e + \frac{\nabla c_{elec}}{c_{elec}} \nabla \psi_e = \frac{3 e_0 a_0^3 \psi_e^2}{c_{elec} e_{au}}\left[E_{WE,abs} - \phi - \Delta\phi_d + \frac{1}{e_0}\left(\mu_{ps} + \mu_{kxc}\right)\right] \tag{S33}$$

where $\mu_{ps} \coloneqq \mu_{ps}^m \Theta(-x) + \mu_{ps}^s \Theta(x)$, $\mu_{kxc} \coloneqq \frac{\partial \varepsilon_{elec}^{kxc}}{\partial n_e}$, and $c_{elec} \coloneqq \frac{a_0^5}{4}\psi_e$.

S3.3. PDEs for species density

The equation for n_i: $\frac{\partial \omega_{tot}}{\partial n_i} = 0$, represents the thermodynamic equilibrium of the solute molecules. This equation is modified as

$$n_i = \frac{1}{V_i} \frac{\gamma_i}{1 + \sum_j \gamma_j} \tag{S34}$$

where $\gamma_i \coloneqq \exp\left[-\frac{1}{k_B T}\left(\frac{\partial \omega_{tot}}{\partial n_i} - \frac{\partial \varepsilon_{mix}^{id}}{\partial n_i} - \frac{V_N}{V_i}\frac{\partial \varepsilon_{mix}^{slv}}{\partial n_i}\right)\right]$.

S4. Additional calculation results

S4.1. Effect of solute-solute interactions in multivalent electrolytes

To confirm the effect of solute-solute interactions, we further ran the calculations in multivalent electrolytes. From the expression of $\mu_{ss,i}$ in Eq. S22, one can expect that the effect of ω_{ss} becomes more significant in higher valence electrolyte. This effect was analyzed by comparing the differential capacitance calculated with the full expression of grand potential (ω_{tot}) and the grand potential without the solute-solute interaction term ($\omega_{tot}^{(7)}$):

$$\omega_{tot}^{(7)} = \omega_{tot} - \omega_{ss}, \quad \text{(S35)}$$

Figure S1 shows the results, showing more significant effect of ω_{ss} in higher valence electrolyte (either $z_a = -2$ or $z_c = 2$) as expected.

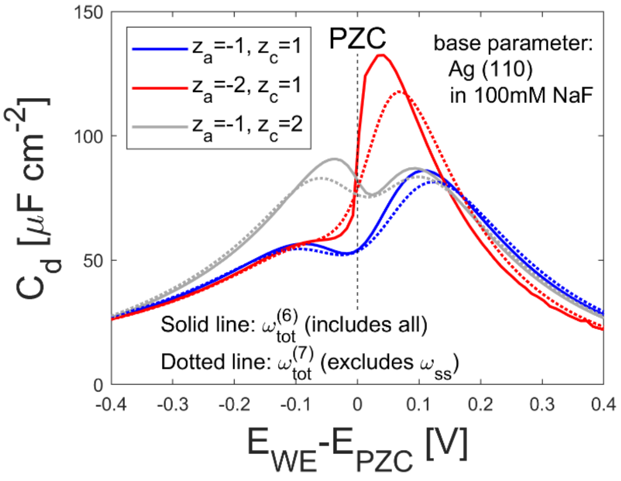

Figure S1. Effect of solute-solute interactions in high valence electrolytes. Solid lines show the results with the full expression of grand potential (ω_{tot}) and the dotted lines show that with the grand potential without the solute-solute interaction term ($\omega_{tot}^{(7)}$). The different colors represent the different valence of electrolyte as shown in the legend. Ion concentrations in the bulk electrolyte is set to satisfy the electroneutrality: c_a^0 = c_c^0 = 100 mM for z_a = -1 and z_c = 1, c_a^0 = 100 mM and c_c^0 = 200 mM for z_a = -2 and z_c = 1, and c_a^0 = 200 mM and c_c^0 = 100 mM for z_a = -1 and z_c = 2.

S4.2. Effect of grand potential components in different order from Section 3.3.

In Section 3.3 of the main text, we analyzed the effect of each grand potential component on the differential capacitance by enabling the component terms one by one as listed in Table 3. The analysis suggested that the polarization energy and size-dependent entropy significantly changed the differential capacitance. The solute-solute interaction was not a significant contributor in the case of monovalent electrolyte, but it can be significant in the case of multivalent electrolyte as shown in Section S4.1. This result, however, can be affected by the order in which the grand potential component is enabled. Here, we confirm the conclusion extracted in Section 3.3 is independent of order.

For this, we disabled each term, rather than enabling them one by one as in Fig. 5 in the main text. In addition to $\omega_{tot}^{(5)}$ in Table 3, which subtracts ω_{mix}^{size} from the total grand potential, and $\omega_{tot}^{(7)}$ in Eq. S35, which subtracts ω_{ss}, another grand potential ($\omega_{tot}^{(8)}$) was defined as a potential without the polarization energy:

$$\omega_{tot}^{(8)} = \omega_{tot} - \omega_{els}^{pol}. \tag{S36}$$

Figure S2 shows that the ω_{mix}^{size} and ω_{els}^{pol} have a significant effect on the differential capacitance, while the effect of ω_{ss} is smaller than those two. This result is consistent with the discussions in Section 3.3 based on the results in Fig. 5.

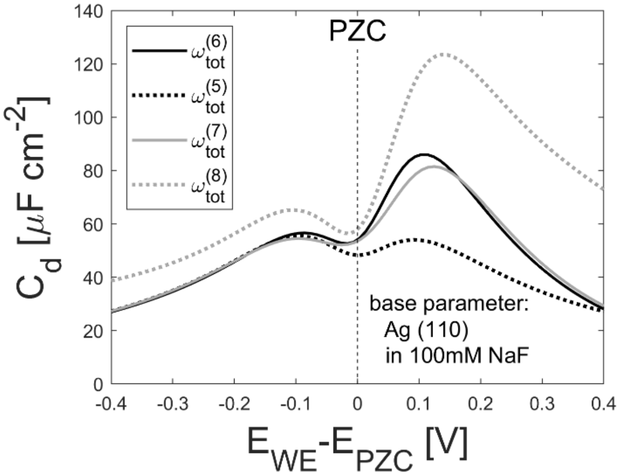

Figure S2. Comparison of calculated differential capacitance with $\omega_{tot}^{(i)}$: The different line styles represent different expressions for ω_{tot} as listed in the legend. $\omega_{tot}^{(6)}$ includes all the interactions, $\omega_{tot}^{(5)}$ excludes the effect of ion-size variation, $\omega_{tot}^{(7)}$ excludes the solute-solute interaction (Eq. S35), and $\omega_{tot}^{(8)}$ excludes the polarization energy (Eq. S36). The parameter set for Ag (110) in 100 mM NaF was used for the calculation. See Table 3 in the main text for the expressions of $\omega_{tot}^{(6)}$ and $\omega_{tot}^{(5)}$.

References

(1) Wu, J. Understanding the Electric Double-Layer Structure, Capacitance, and Charging Dynamics. *Chem Rev* **2022**, *122* (12), 10821-10859. DOI: 10.1021/acs.chemrev.2c00097.

(2) Huang, J. Density-Potential Functional Theory of Electrochemical Double Layers: Calibration on the Ag(111)-KPF(6) System and Parametric Analysis. *J Chem Theory Comput* **2023**, *19* (3), 1003-1013. DOI: 10.1021/acs.jctc.2c00799.

(3) Smith, J. R. Self-Consistent Many-Electron Theory of Electron Work Functions and Surface Potential Characteristics for Selected Metals. *Physical Review* **1969**, *181* (2), 522-529. DOI: 10.1103/PhysRev.181.522.

(4) Dhattarwal, H. S.; Gao, A.; Remsing, R. C. Dielectric Saturation in Water from a Long-Range Machine Learning Model. *J Phys Chem B* **2023**, *127* (16), 3663-3671. DOI: 10.1021/acs.jpcb.3c00390.

(5) Bikerman, J. J. XXXIX. Structure and capacity of electrical double layer. *The London, Edinburgh, and Dublin Philosophical Magazine and Journal of Science* **2009**, *33* (220), 384-397. DOI: 10.1080/14786444208520813.

(6) Perdew, J. P.; Tran, H. Q.; Smith, E. D. Stabilized jellium: Structureless pseudopotential model for the cohesive and surface properties of metals. *Phys Rev B Condens Matter* **1990**, *42* (18), 11627-11636. DOI: 10.1103/physrevb.42.11627.

(7) Ashcroft, N. W. Electron-ion pseudopotentials in metals. *Physics letters* **1966**, *23* (1), 48-50.
